# Ethical and coordinative challenges in setting up a national cohort study during the COVID-19 pandemic in Germany

**DOI:** 10.1186/s12910-023-00959-0

**Published:** 2023-10-17

**Authors:** Katharina Tilch, Sina M. Pütz, Katharina Appel, Monika Kraus, Bettina Lorenz-Depiereux, Lisa Pilgram, Gabi Anton, Sarah Berger, Ramsia Geisler, Kirsten Haas, Thomas Illig, Dagmar Krefting, Roberto Lorbeer, Lazar Mitrov, Maximilian Muenchhoff, Matthias Nauck, Christina Pley, Jens-Peter Reese, Siegbert Rieg, Margarete Scherer, Melanie Stecher, Christoph Stellbrink, Heike Valentin, Christof Winter, Martin Witzenrath, J. Janne Vehreschild

**Affiliations:** 1 Faculty of Medicine, Department I of Internal Medicine, Center for Integrated Oncology, Aachen Bonn Cologne Duesseldorf, University of Cologne, University Hospital Cologne, Cologne, Germany; 2 Department of Internal Medicine, Hematology/Oncology, Goethe University Frankfurt, Frankfurt am Main, Germany; 3 Helmholtz Center Munich, Institute of Epidemiology, Research Unit Molecular Epidemiology, Munich, Germany; 4 German Center for Cardiovascular Research (DZHK), Partner Site Munich, Munich, Germany; 5 Department of Nephrology and Medical Intensive Care, Charité - Universitätsmedizin Berlin, Berlin, Germany; 6 German Centre for Infection Research (DZIF), Partner Site Munich, Munich, Germany; 7 Charité - Universitätsmedizin Berlin, Corporate Member of Freie Universität Berlin and Humboldt-Universität Zu Berlin, Department of Infectious Diseases, Respiratory Medicine and Critical Care, Berlin, Germany; 8 Institute of Clinical Epidemiology and Biometry, University of Würzburg, Julius Maximilian University of Würzburg, Würzburg, Germany; 9 University Hospital Würzburg, Institute for Medical Data Science (ImDS), Josef-Schneider Straße 2, 97080 Würzburg, Germany; 10 Hannover Unified Biobank, Hannover Medical School, Hannover, Germany; 11 Department of Medical Informatics, University Medical Center Göttingen, Göttingen, Germany; 12 Deutsches Herzzentrum der Charité, Medical Heart Center of Charité and German Heart Institute Berlin, Institute of Computer-Assisted Cardiovascular Medicine, Berlin, Germany; 13 Department of Radiology, University Hospital LMU Munich, Munich, Germany; 14 Max Von Pettenkofer Institute & GeneCenter, Virology, Faculty of Medicine, Ludwig-Maximilians University, Munich, Germany; 15 Institute of Clinical Chemistry and Laboratory Medicine, University Medicine Greifswald, Greifswald, Germany; 16 Division of Infectious Diseases, Department of Medicine II, Medical Centre - University of Freiburg, Faculty of Medicine, University of Freiburg, Freiburg, Germany; 17 German Center for Infection Research (DZIF), Partner-Site Cologne-Bonn, Cologne, Germany; 18 Bielefeld University, Medical School and University Medical Center East Westphalia-Lippe, Klinikum Bielefeld, Academic Department of Cardiology and Internal Intensive Care Medicine, Bielefeld, Germany; 19 Trusted Third Party of the University Medicine Greifswald, Ellernholzstr. 1-2, 17475 Greifswald, Germany; 20 School of Medicine, Institute of Clinical Chemistry and Pathobiochemistry, Technical University of Munich, Munich, Germany; 21 TranslaTUM, Center for Translational Cancer Research, Technical University of Munich, Munich, Germany; 22 German Center for Lung Research (DZL), Berlin, Germany; 23 Department I for Internal Medicine, Faculty of Medicine, University Hospital of Cologne, University of Cologne, Cologne, Germany

**Keywords:** Pandemic preparedness, Ethical approval, COVID-19, Multicenter study, Study initiation, Ethics committee

## Abstract

With the outbreak of severe acute respiratory syndrome coronavirus 2 (SARS-CoV-2), global researchers were confronted with major challenges. The German National Pandemic Cohort Network (NAPKON) was launched in fall 2020 to effectively leverage resources and bundle research activities in the fight against the coronavirus disease 2019 (COVID-19) pandemic. We analyzed the setup phase of NAPKON as an example for multicenter studies in Germany, highlighting challenges and optimization potential in connecting 59 university and nonuniversity study sites. We examined the ethics application process of 121 ethics submissions considering durations, annotations, and outcomes. Study site activation and recruitment processes were investigated and related to the incidence of SARS-CoV-2 infections. For all initial ethics applications, the median time to a positive ethics vote was less than two weeks and 30 of these study sites (65%) joined NAPKON within less than three weeks each. Electronic instead of postal ethics submission (9.5 days (Q1: 5.75, Q3: 17) vs. 14 days (Q1: 11, Q3: 26), *p* value = 0.01) and adoption of the primary ethics vote significantly accelerated the ethics application process. Each study center enrolled a median of 37 patients during the 14-month observation period, with large differences depending on the health sector. We found a positive correlation between recruitment performance and COVID-19 incidence as well as hospitalization incidence. Our analysis highlighted the challenges and opportunities of the federated system in Germany. Digital ethics application tools, adoption of a primary ethics vote and standardized formal requirements lead to harmonized and thus faster study initiation processes during a pandemic.

**Supplementary Information:**

The online version contains supplementary material available at 10.1186/s12910-023-00959-0.

## Introduction

Studying coronavirus disease 2019 (COVID-19) as a rapidly evolving disease with a heterogeneous mix of short- and long-term sequelae in the context of continuously changing population immunity and treatment options remains a major challenge [1, 2]. Comprehensive cohort studies need to obtain sufficient case numbers to be representative of various risk groups, virus variants, immunization statuses, and treatment approaches and need to follow patients throughout their course of disease as they shift between treatment centers and health care sectors. Thus, large networks of care providers from different health care sectors and disciplines need to be involved [1].

However, research in a large network requires versatile infrastructures and a high level of trust and engagement by all stakeholders. From December 2019 to December 2021, more than 3,000 single- and multicenter observational cohort studies related to COVID-19 were established worldwide [2], leading to a rapid increase in knowledge [3–6] within a short time. The findings directly influenced public and political decision-making. While each study addressed important aspects of COVID-19, a minority of the studies followed structured, harmonized and quality-controlled data and biosample acquisition methods across all health care sectors to cover the entire spectrum of the disease. Projects of this scale were, for example, the CANCOV [7, 8], SARS-Brazil [9], FrenchCOVID [10], and ISARIC Registry studies [11]. Several clinical trials were able to build on existing national structures, whereas in Germany, there was no existing research network that would ensure a harmonized study approach across all university hospitals. Therefore, in March 2020, the Federal Ministry of Education and Research (BMBF) funded the Network University Medicine (NUM) to enable national research in the context of the COVID-19 pandemic and beyond. One of the key projects within NUM is the German National Pandemic Cohort Network (NAPKON), a prospective cohort study that has been recruiting laboratory-confirmed severe acute respiratory syndrome coronavirus 2 (SARS-CoV-2)-positive patients from more than 50 inpatient and outpatient study sites across Germany since November 2020 [12].

In Germany, research on a national level faces several major challenges. In particular, challenging and time-consuming legal and regulatory processes are blamed for causing a competitive disadvantage to the German research community [13–15]. To our knowledge, there are no published analyses on the challenges German research centers face in initiating and implementing clinical epidemiological studies on a national level. Comprehensive catalogues of requirements and checklists for setting up cohort studies were proposed as supporting tools for researchers [16, 17] but need evaluation against actual processes and tangible examples. The extensive planning that precedes a prospective, longitudinal, multicenter study and the ongoing re-evaluation of requirements during the study period were described by Patuleia et al. [18], and the challenges in obtaining ethics votes and recruiting patients for an international clinical registry were described by Kates et al. [19]. They highlighted that standardized procedures for the preparation of study protocols and standard operating procedures (SOPs) are often lacking for multicenter studies. The mentioned studies further noted that the process of ethics approval and study site initiation is time-consuming and often leads to delays in patient recruitment. However, setting up a study such as NAPKON duringa pandemic — when the entire science community is put to the test — has never been systematically described before.

In this article, we systematically evaluated the setup and initiation processes of the nationwide cohort study NAPKON as a national effort involving all university hospitals and many nonacademic study sites. Our analysis provides information on important aspects of the research infrastructure in Germany and can be used as a showcase setup for other comprehensive cohort studies. We focused on the aspects of ethical approval, patient recruitment, and patient consent in the initiation process of NAPKON and noted characteristics of the federal system in Germany.

## Materials and methods

### NAPKON Cohorts

As previously described in detail by Schons et al. [12], NAPKON is divided into three complementary cohorts: the Cross-Sectoral Platform (*Sektorenübergreifende Plattform,* SUEP), the High-Resolution Platform (*Hochauflösende Plattform,* HAP) and the Population-Based Platform (*Populationsbasierte Plattform,* POP). Since the POP recruits at three university hospitals and follows an already established protocol, our analysis focused on the SUEP and HAP. Germany has 38 university and many nonuniversity hospitals [20]. The SUEP recruits SARS-CoV-2 patients and controls at 28 university and 20 nonuniversity study sites and outpatient practices following a detailed study protocol; aiming for a different subgroup of SARS-CoV-2 patients, the HAP recruits at 11 selected university hospitals [12, 21] following a more comprehensive study protocol compared to the SUEP. The first patient enrolled in NAPKON was recruited in the SUEP on November 4, 2020. Our analysis focused on the roll-out of NAPKON from the beginning of the ethics application process to the end of the first funding period, concluding on December 31, 2021. The submission of the initial ethics applications for the SUEP and HAP study sites marked the beginning of the NAPKON roll-out, as this event defined the completion of internal preparatory steps.

### Ethics procedure and study site activation

#### Ethical consultation for physicians

**Fig. 1:**
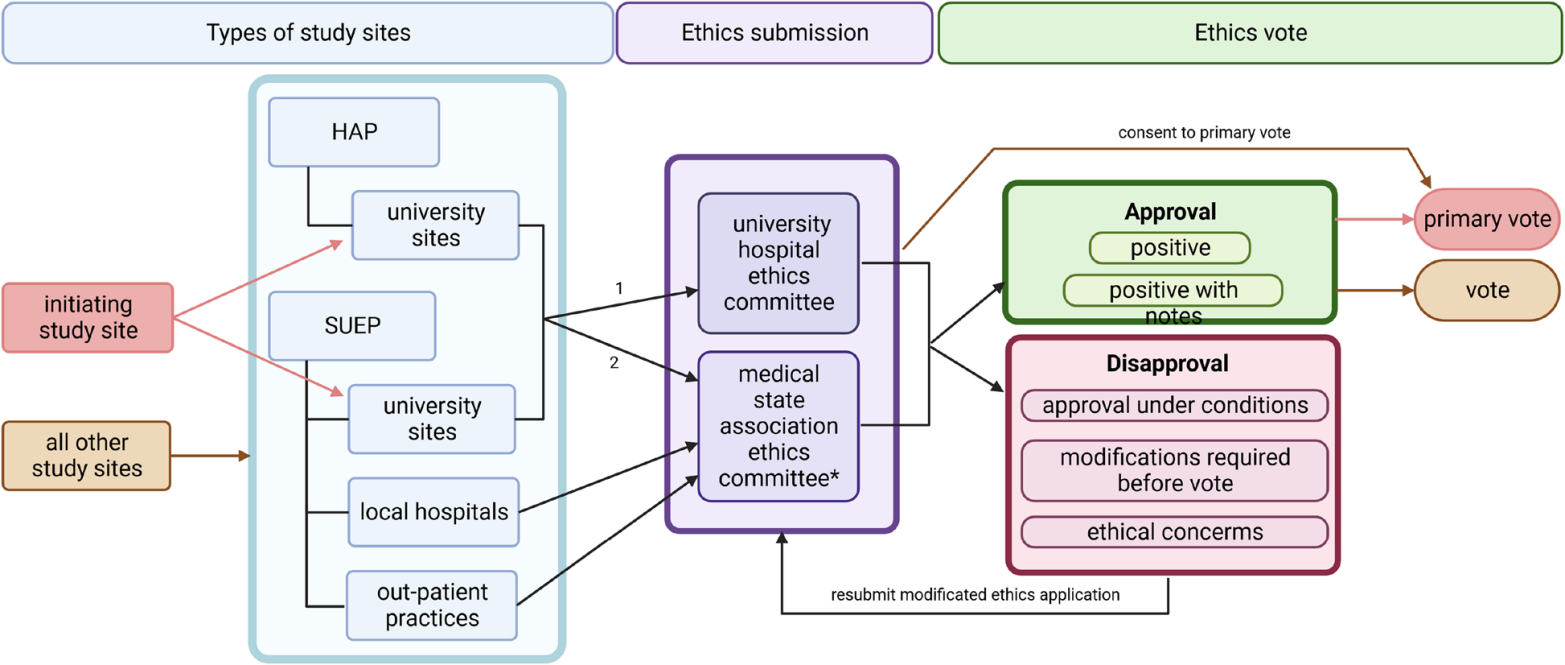
Ethics application process in NAPKON. *Germany has 17 medical state associations with specific regulations for the ethical consultation of physicians. Some ethics committees perform an in-depth review of each new study, while others accept the ethical approval of the primary ethics committee [23]. Bavaria is the only one not to require further ethics applications after the primary vote [24] ^1^ with own university ethics committee; ^2^ without own university ethics committee; SUEP = Cross-Sectoral Platform; HAP = High-Resolution Platform. Created with BioRender.com

In Germany, ethics committees classify clinical studies by their type of intervention, and specific regulatory rules apply accordingly. The NAPKON project, as a prospective, observational cohort with no experimental diagnostic or treatment arm, classifies as “other” medical research that is not regulated by specific legislation relating to medical devices or drugs. All physicians taking part in the study must be ethically advised by a responsible ethics committee, as specified by their professional code of conduct [22]. After ethics approval at one study site, new physicians at this site can be subsequently advised under the existing ethics vote. Responsible ethics committees vary depending on the health care sector (Fig. 1). In the following, we defined the positive outcome of ethics consultations as “approval” to allow for a uniform evaluation.

#### Ethics application

In Germany, ethical guidance for cohort studies taking place at multiple study sites follows the concept of the initiating study site receiving a primary vote and subsequent study sites referring to this vote. This ensures efficient coordination of the ethics applications and identification of any issues in the protocol and study-related documents before the nationwide roll-out. An ethics coordination team supported the initiating study site in preparing, reviewing, and adapting the ethics application documents, such as each cohort’s study protocol, patient information documents and consent forms. The documents were carefully reviewed for ethical or data protection information.

Subsequent substantial changes to the originally consulted study documents must be reviewed again by an ethics committee. For this, study-specific so-called *amendments* were resubmitted.

The submission process for ethics applications and amendments is described in Fig. 1, and the application process for amendments followed the same routine as that from the initial ethics votes. In the following, we referred to the submissions resulting in the first approving vote from an ethics committee as the initial submission. The initial submission also applied to study sites joining the study later with documents already containing amendments. The whole process, from submission to approval, is referred to as an application process for ethical approval (ethics application).

#### Review of ethics annotations

The annotations of the ethics committees for the submitted study documents (Table S1) of the SUEP and HAP were analyzed with regard to document type, type of request and thematic content. We defined four categories for the type of request: content (e.g., to clarify the deferred consent procedure), formal (e.g., to emphasize text passages in bold letters), comprehensibility (e.g., to avoid technical vocabulary, understandable for laymen) and resubmission of documents (e.g., an insurance certificate). For the detailed analysis of the content-related annotations, each content request was assigned one of 147 keywords, and each keyword was in turn categorized into one of six topics. For example, the annotation "It is not apparent from the submitted documents and study protocol, why genetic testing up to complete genome sequencing is necessary for the study." was tagged with the keyword "genetic testing" and assigned to the category "biosample collection". The analysis was conducted in German independently by two authors. Only the categories were translated to English prior to publication.

All ethics votes were collected from the study sites and filed by the SUEP and HAP teams using the NAPKON online cloud service. Paper votes were either scanned and filed or collected and handed over in person. We obtained access to the relevant folders in the cloud and to two online ethics portals (ethikPool) [25]. Email conversations relevant to the roll-out process were also provided. All votes received until the end of 2021 were considered.

#### Study site activation

In general, ethical approval of the study and the responsible physician is a prerequisite for activation of the productive versions of the data platforms. This prevents the study staff from including patients in the study before ethical approval. Activating a study site requires registering the study staff and all devices in the central data platforms and training them using the data platforms and conduct the study. Activation time was defined as the interval from ethics approval until complete activation.

### Recruitment

Recruitment numbers were determined based on the number of informed consent documents, including those from patients who later withdrew their consent. When consenting participate in the study, patients were asked to agree or disagree with a number of specific options depending on the platform and health care sector. For example, patients could agree or disagree on recontacting options or further examinations (see the Results section for more details). Although the SUEP study protocol included biosample collection in principle for all study centers, in some cases, this was waived in favor of participation [12].

For activation and recruitment information, we requested dates and informed consent data from the trusted third party in accordance with patient data protection and with permission from the platform coordinators. To compare the heterogeneity of the study site recruitment within their respective platforms or sectors, we defined the categories of low- and high-performing sites. A high-performing site recruited a particularly high number of patients relative to all included patients on their platform (fourth quartile), while a low-performing site recruited a relatively small number of patients (first quartile).

### Statistical analysis

The data were processed using Microsoft® Excel® (Version 2212 Build 16.0.15928.20196, 2018, Microsoft Corporation, Redmond, Washington, USA, https://office.microsoft.com/excel) and analyzed using RStudio (Version 2022.7.2.576, Integrated Development Environment for R. RStudio, PBC, Boston, MA, http://www.rstudio.com/). Descriptions of the parameters regarding the ethics votes and patient recruitment are represented as absolute numbers and percentages. Time durations during the ethics application process were counted in days and presented as medians and first and third quartiles. Application processing time according to the type of application and time until positive ethics vote according to the number of annotations were visualized with grouped boxplots. Statistical significance was shown using the Mann‒Whitney U test or log-rank test, as appropriate, with a *p* value < 0.05 as the significance level. The strength and direction of linear correlations were calculated using the Pearson correlation coefficient. The average (hospitalization) incidence per calendar week was calculated using data from the Robert Koch Institute (RKI) [26]. We used the pandemic wave description from the epidemiological bulletin 10/2022 of the RKI to classify the pandemic into different waves [27].

## Results

### Initial ethics application process

**Fig. 2:**
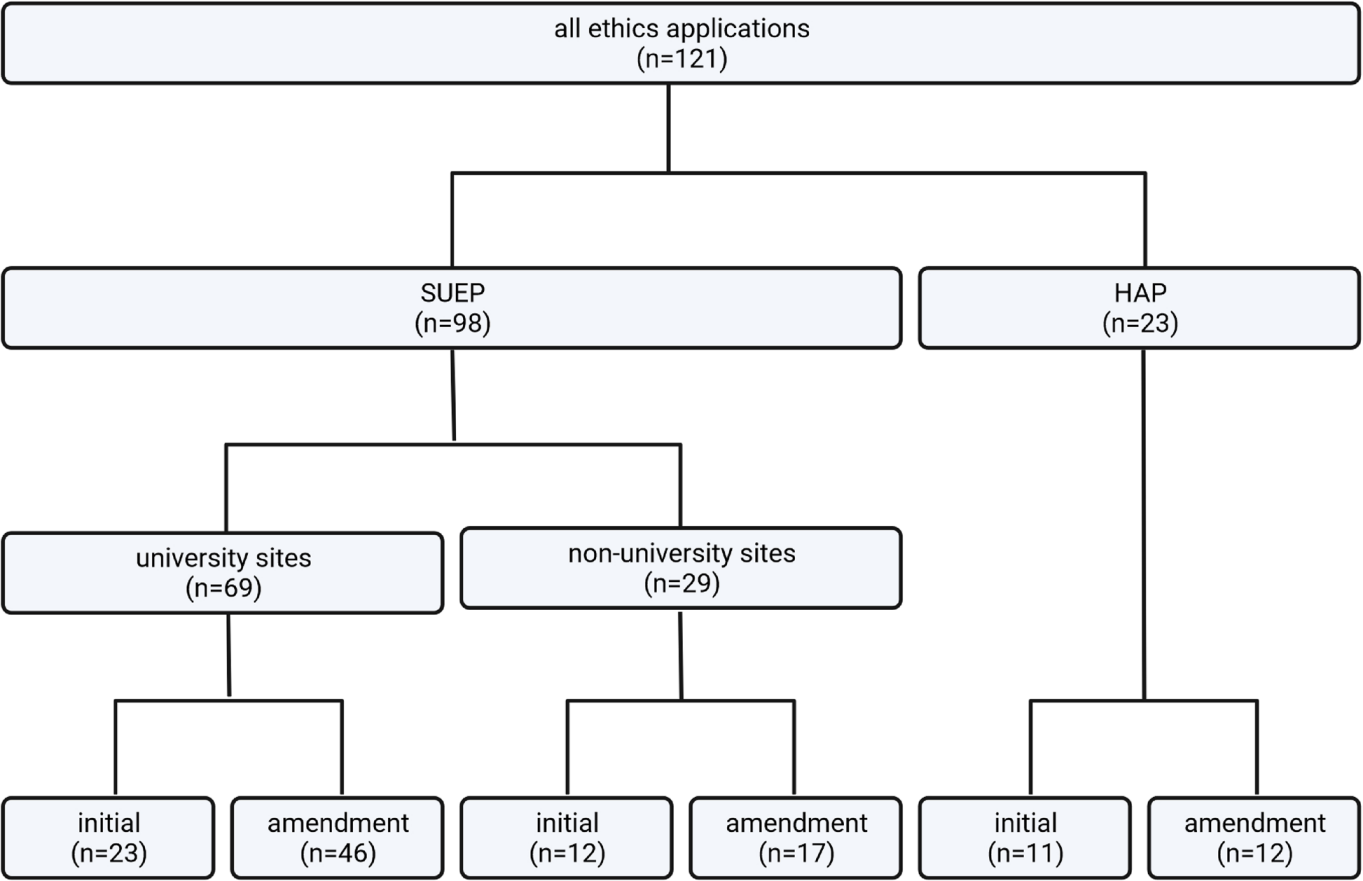
NAPKON ethics application overview. The organigram shows all considered ethics applications divided by platform, health care sector and type of application. SUEP = Cross-Sectoral Platform; HAP = High-Resolution Platform. Created with BioRender.com

The SUEP received the primary approving vote on November 3, 2020, from the Ethics Committee of the Department of Medicine at Goethe University Frankfurt (local ethics ID approval 20–924) and the HAP received the primary approving vote on October 29, 2020, from the Ethics Committee of the Charité – Universitätsmedizin Berlin (local ethics ID approval EA2/066/20 and EA2/226/21). By the end of 2021, four substantial amendments were implemented in the SUEP and two in the HAP. In total, 121 ethics votes were received (Fig. 2), and 353 ethics annotations were recorded and analyzed.

**Fig. 3:**
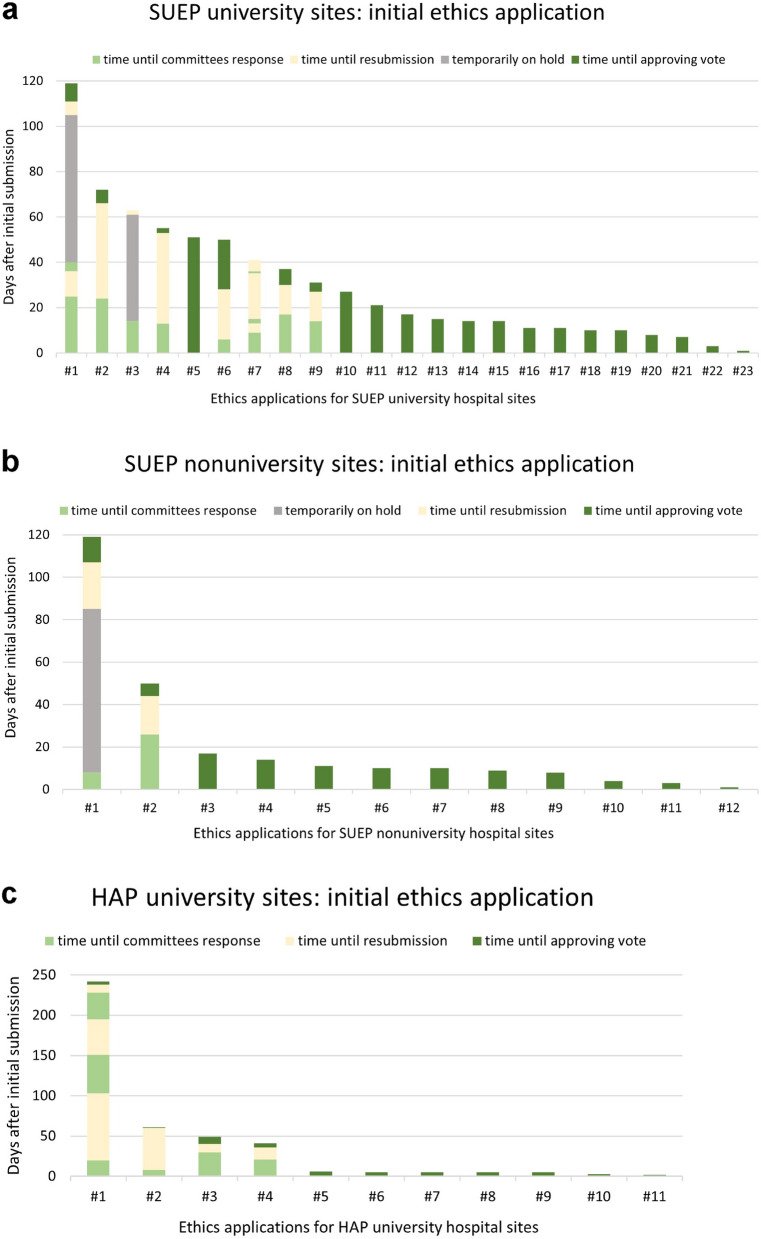
Time of initial ethics application processes. Applications are numbered and sorted according to the total time of the initial ethics application process. Initial ethics application processes are described for (**a**) 23 Cross-Sectoral Platform (SUEP) applications for university hospital ethics committees, (**b**) 12 SUEP applications for state medical association ethics committees, and (**c**) 11 High-Resolution Platform (HAP) ethics applications for university hospital ethics committees. The initial ethics vote of a state medical association applied in each case to the first nonuniversity site in the respective state. Ethics applications were submitted to 12 state medical associations. The following sites joined the vote

Having received the primary votes at the initiating study sites in Frankfurt and Berlin, 23 of 30 university hospitals participating in the SUEP (77%) submitted their initial study documents for ethical consultation to the ethics committee of the respective university hospital (Fig. 3a). The remaining seven university study sites consulted the ethics committee of the respective state medical association. For simplicity, we considered and listed them together with the nonuniversity study sites (Fig. 3b).

The median time from submission to receiving a positive vote was 17 days (first quartile (Q1): 10, third quartile (Q3): 45.5) for all SUEP university study sites, with one outlier reaching 119 days. In eight cases (35%), the initial vote was not approving, modifications to the applications were required or conditions had to be met (Fig. 3a, study sites Nos. 1–4 and 6–9). The median processing time for fulfilling the claims by the SUEP initiating study sites’ team was 19.5 days (Q1: 13, Q3: 31.75), excluding time spent waiting for other votes or documents to comply. Time from final resubmission of the modified documents until receipt of an approving vote took a median of five days (Q1: 1.5, Q3: 7.25). All SUEP university study sites finally received a positive ethics vote.

The consultation for the 12 state medical associations’ ethics committees took a median time of ten days (Q1: 7, Q3: 14.75) until final the vote, with a maximum time of 119 days to be emphasized (Fig. 3b). The median time until the first response was 9.5 days (Q1: 7, Q3: 11.75), which was approving in ten applications (83%). For two applications (17%), the initial vote was not approving, with a median processing time for modifications of 20 days and a median response time after resubmission of nine days (Fig. 3b, study sites Nos. 1 and 2).

Since HAP study sites were all university hospitals, ethics consultations took place with their corresponding ethics committees. Figure 3c shows the initial application processes of the 11 HAP study sites: the median time until the first response as well as the final ethics vote was five days (Q1: 5, Q3: 45), with an outlier reaching 242 days until approval. In four cases (36%), the first votes were not approving, and the median condition processing time was 33.5, days while the median response time after the last resubmission was 4.5 days (Fig. 3c, study sites Nos. 1–4).

### Ethical amendment application process

In total, there were 75 submissions for amendments in the SUEP and HAP. The median overall processing time by the responsible ethics committees was 11 days (Q1: 6, Q3: 23). The amendment application process was faster for the state medical association ethics committees of the SUEP, with a median time of ten days, followed by the university hospital ethics committees of the SUEP, with a median time of 11 days and the university hospital ethics committees of the HAP, with a median time of 17 days.

### Types of ethics submissions

**Fig. 4:**
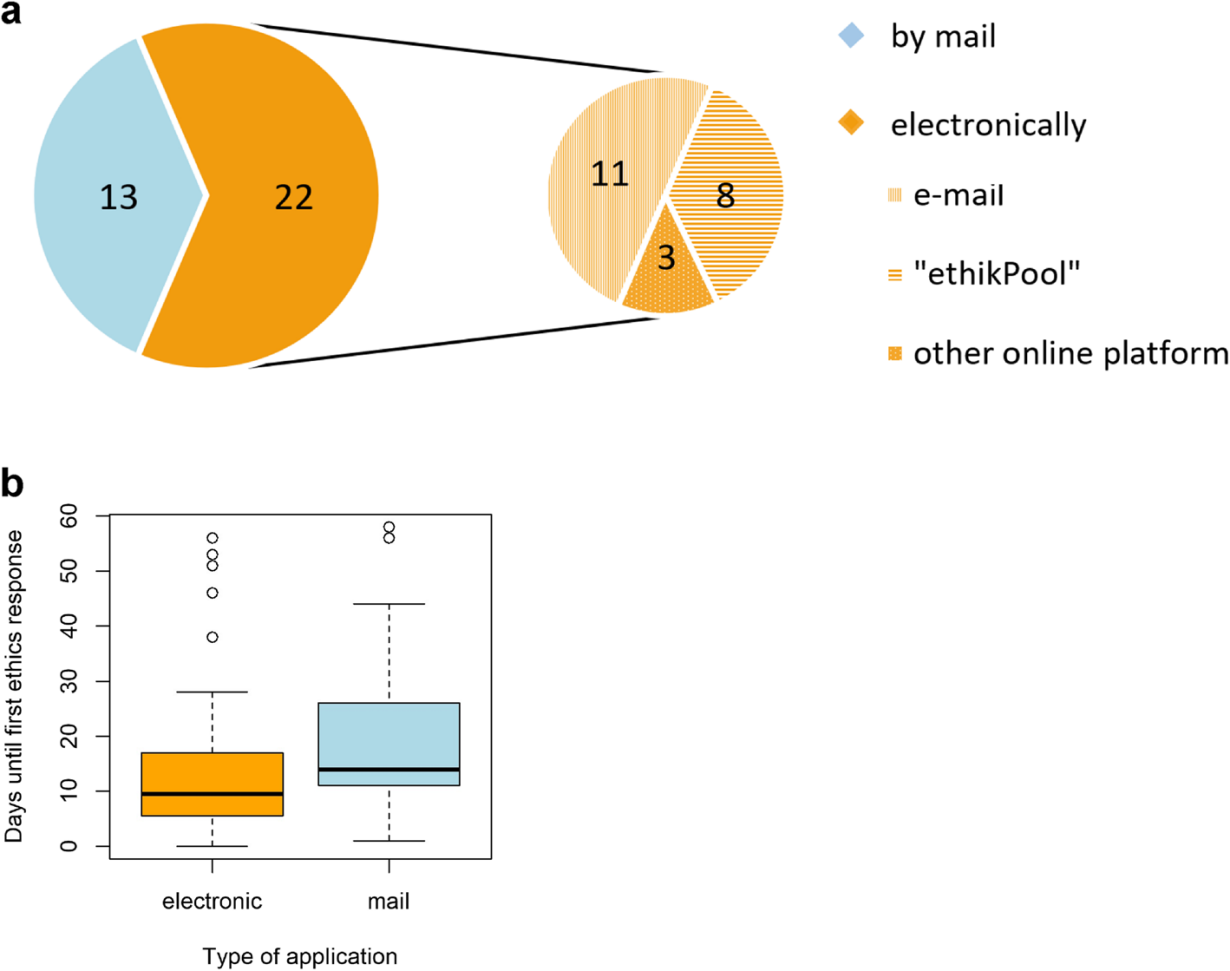
Submission types for ethical approval. (**a**) Submission types for initial ethics applications at 35 university and state medical association ethics committees of the Cross-Sectoral Platform (SUEP) stated in absolute numbers. (**b**) Ethics application processing time according to the type of application (electronic or mail). Thirty-five initial applications and 63 amendment applications of the SUEP were considered (*n* = 98). Durations are shown in days from submission until the first response of the ethics committees

Most of the initial applications for ethical consultation for the university and nonuniversity SUEP study sites were submitted electronically (email, upload to web-based submission platform (*ethikPool*) [25], or upload to another online ethics submission platform), while fewer were submitted by mail (if at least one document was requested to be sent by mail) (Fig. 4a).

Figure 4b illustrates the average time taken by the ethics committees to first reply stratified by type of application of the SUEP study sites. The median response time for electronic applications was 9.5 days (Q1: 5.75, Q3: 17), while mail applications took a median of 14 days (Q1: 11, Q3: 26). A significant difference (*p* value = 0.01) in the time until first response between mail and electronic applications was calculated for all 98 SUEP applications including initial ethics applications and amendments, using the Mann‒Whitney U test.

### Consent for primary vote

We found that at the university level, the responsible ethics committees of the SUEP and HAP adopted the primary vote for 33 submissions (36%), while 49 (53%) underwent the regular review process. Ten votes (11%) belonging to the primary voting ethics committees were excluded. At the state medical association level, the ethics committees adopted the primary vote in 20 cases (69%) and chose the regular application process in nine cases (31%). Adopting a primary vote resulted in time savings of six days (median 11 vs. 17 days) for university and three days (median 9 vs. 12 days) for nonuniversity study sites.

### Ethics vote outcomes

The overall time for an ethics application process depends on the outcome of the vote and the annotations made by the ethics committees. For the majority of the SUEP (*n* = 26, 74%) and HAP (*n* = 7, 64%) initial submissions, the ethics committees approved directly (positive or positive with notes) (Table 1). For amendment submissions, the direct acceptance rate was even higher. Nonapproved votes for the SUEP were equally frequent for state medical association ethics committees (*n* = 3, 10%) and university hospital ethics committees (*n* = 10, 17%) (data not shown in the table). All applications that were not approved were resubmitted and received ethical approval after major revisions.

**Table 1:** Outcome of the (a) Cross-Sectoral Platform (SUEP) ethics votes of the initial application process (*n* = 35) and the amendment applications (*n* = 63) as well as both processes (= All, *n* = 98) and that of the (b) High-Resolution Platform (HAP) ethics votes of the initial application process (*n* = 11) and the amendment applications (*n* = 12) as well as both processes (= All, *n* = 23). Data are shown as absolute numbers and percentages of the corresponding total number of ethics votes

**a**			
**Ethics vote outcome SUEP**	**Initial; n (%)**	**Amendment; n (%)**	**All; n (%)**
Positive	16 (46)	47 (75)	63 (64)
Positive with notes	10 (29)	12 (19)	22 (22)
Approval under conditions	6 (17)	1 (2)	7 (7)
Modifications required before vote	3 (9)	2 (3)	5 (5)
Ethical concerns	0 (0)	1 (2)	1 (1)
**b**			
**Ethics vote outcome HAP**	**Initial; n (%)**	**Amendment; n (%)**	**All; n (%)**
Positive	6 (55)	11 (92)	17 (74)
Positive with notes	1 (9)	0 (0)	1 (4)
Approval under conditions	3 (27)	1 (8)	4 (17)
Modifications required before vote	1 (9)	0 (0)	1 (4)
Ethical concerns	0 (0)	0 (0)	0 (0)

### Application processing time according to number of annotations

We found a positive correlation between the number of annotations in an ethics application and the overall time to receipt of approval for ethics applications: the ethics application time increased with the number of annotations (Pearson product-moment correlation *r* = 0.52; *p* value < 0.001).

**Fig. 5:**
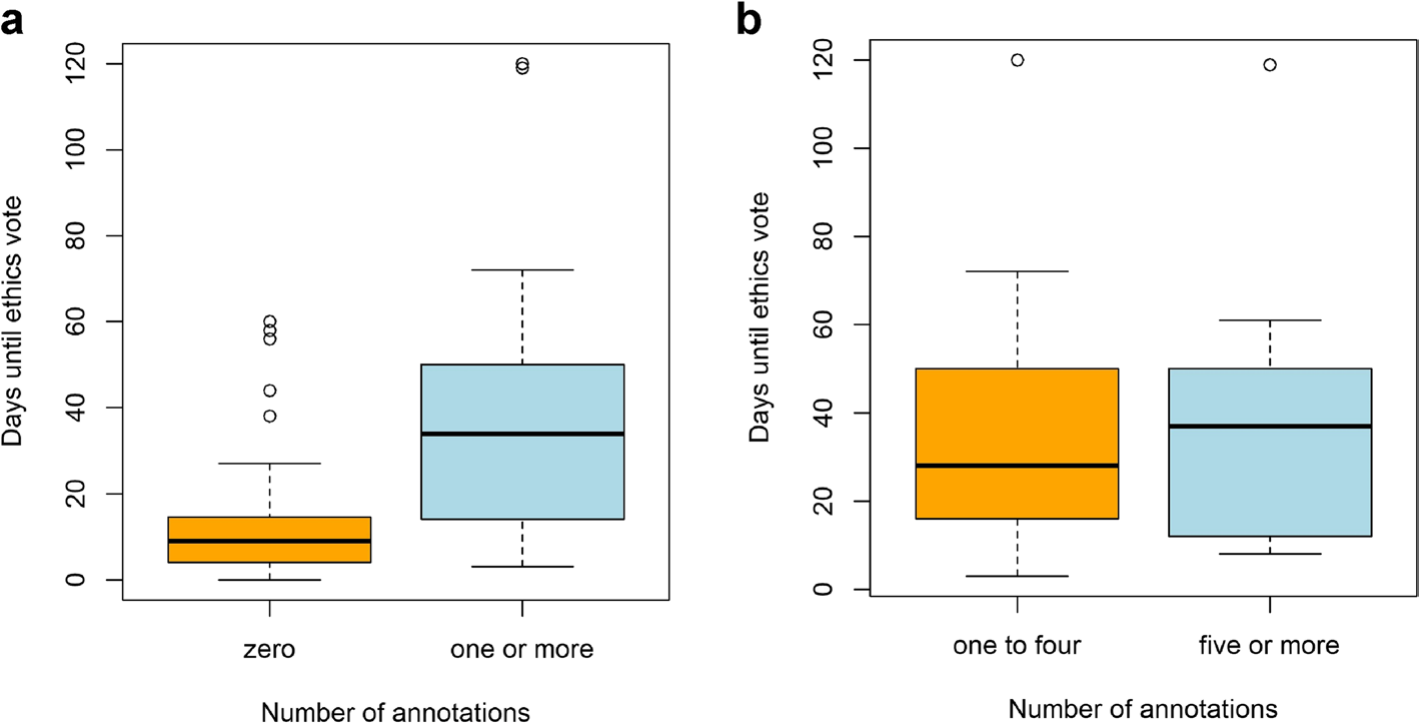
The time until a final ethics vote was received for the Cross-Sectoral Platform (SUEP) and the High-Resolution Platform (HAP) study sites (*n* = 120) is shown in days with regard to the number of annotations: (**a**) zero (*n* = 80) vs. one or more (*n* = 40) annotations, (**b**) one to four (*n* = 21) vs. five or more (*n* = 19) annotations. For better visualization, the HAP study site outlier with 242 days of ethics process time was not included. Analysis containing this study site showed comparable significance levels

To determine the resulting time difference, we grouped the votes according to the number of annotations. We found that the time until ethic committee response was significantly longer (log-rank test: *p* value < 0.001) if at least one annotation was made by the ethics committee in comparison to zero annotations (Fig. 5a). Dividing the group with at least one annotation into two roughly equal groups, there was no significant difference (log-rank test: *p* value = 1) in ethics application processing time when one to four compared to five or more annotations were made (Fig. 5b).

### Annotations

Approximately half of the annotations made by the ethics committees addressed the patient information document (*n* = 186, 53%), and one-fifth addressed consent forms (*n* = 73, 21%), with similar results for the SUEP and the HAP.

Other frequently addressed documents were the study protocol (*n* = 54, 15%), documents on data privacy (*n* = 11, 3%) and the terms of use of the NUM (*n* = 5, 1%). Some annotations were categorized as “others”, as they concerned all study documents submitted or addressed very specific documents (*n* = 25, 7%).

Considering the ethics votes of the SUEP and HAP together, content requests were the most frequent types of annotations (*n* = 147, 42%). In combination with formal requests (*n* = 121, 34%), they accounted for three-quarters of all annotations. Annotations requesting improved comprehensibility (*n* = 51, 14%) and further documents (*n* = 34, 10%) were less frequent. SUEP and HAP votes showed similar distributions of the different types of requests. For the HAP, formal requests (*n* = 45, 39%) were slightly more frequent than content requests (*n* = 43, 37%). For votes from state medical associations, the second most frequent type of annotation was requests to improve the comprehensibility of the documents (*n* = 15, 29%).

In total, 147 keywords were screened, whose distribution among the six main topics is shown in Table 2. Most of the annotations were made on the topic "patient information and consent" in the SUEP and on the topic "biosample collection" in the HAP. In both platforms, these two topics accounted for more than half of the annotations.

**Table 2:** Thematic focus of content requests for keywords in Cross-Sectoral Platform (SUEP, *n* = 104) and High-Resolution Platform (HAP, *n* = 43) ethics votes. Six categories were made that summarized the thematic focus. Data show the absolute number of content requests for one topic and the relative share of this thematic focus in a column

Category	HAP; n (%)	SUEP; n (%)	All; n (%)
Framework of the study	7 (16)	18 (17)	25 (17)
Patient information and consent	9 (21)	36 (35)	45 (31)
Study procedures	8 (19)	9 (9)	17 (12)
Data processing	5 (12)	6 (6)	11 (8)
Biosample collection	10 (23)	22 (21)	32 (22)
Secondary use and use abroad	4 (9)	13 (13)	17 (12)

### Study site activation

**Fig. 6:**
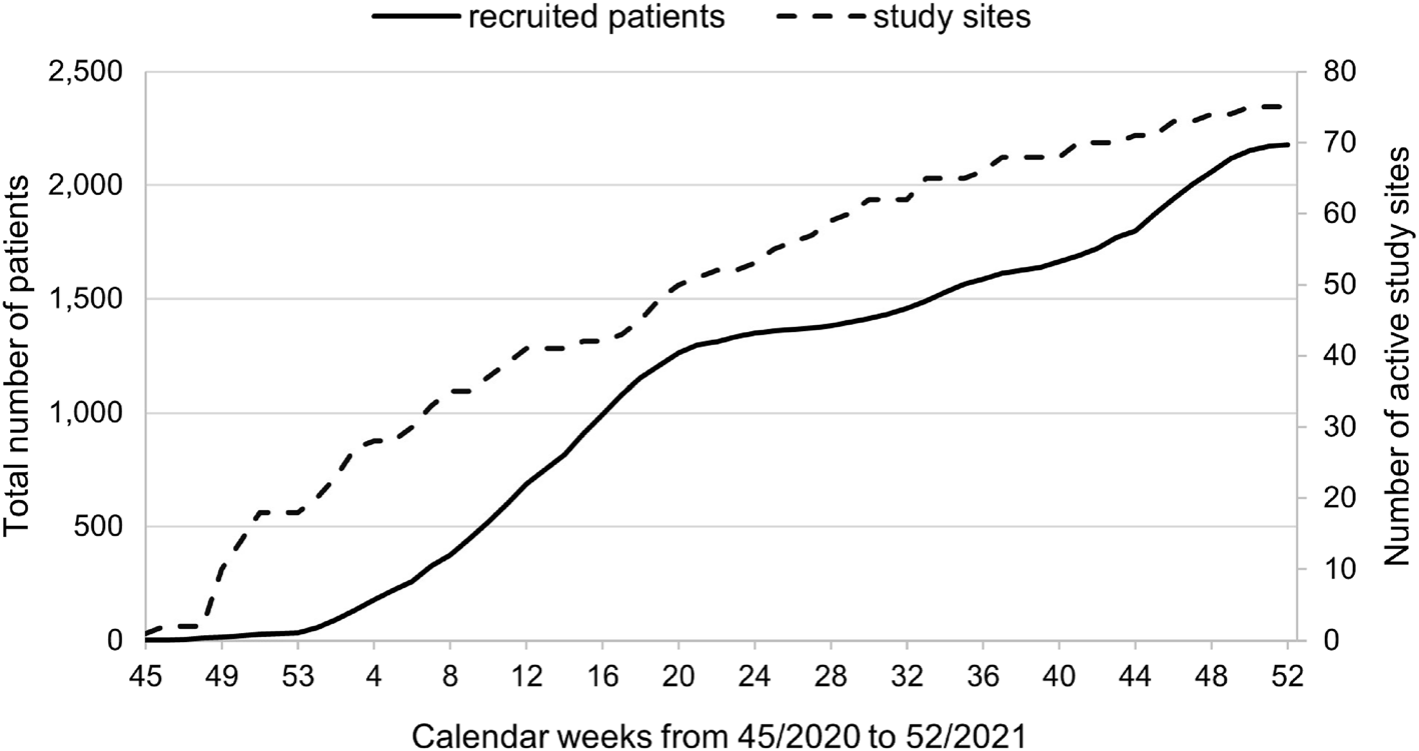
Development of the number of activated study sites and patient recruitment in the Cross-Sectoral Platform (SUEP) and High-Resolution Platform (HAP). The number of recruited patients and activated study sites was added weekly and the time was given in calendar weeks of the period observed

The number of activated study sites in both cohorts increased rapidly at the beginning of the study; half of them had already joined NAPKON by March 2021 (Fig. 6, calendar week ten (2021)). For four study sites, activation times were negative and excluded from the evaluation because these study sites were activated before an approving ethics vote was received. The reasons were as follows: a letter of clearance issued in advance by the ethics committee; active access mistakenly set up instead of test access and study sites expecting a positive vote, but formal matters still needing to be settled. While the median time for study site activation after a positive ethical vote was 11 days (Q1: 5, Q3: 25.5), the median time from activation until the first patient was recruited exceeded five weeks (38 days, Q1: 14.75, Q3: 62). The median time from ethics approval until the first patient was recruited was 54 days (Q1: 35, Q3: 82.5), with the shortest duration of one day and the longest duration of 170 days.

Focusing on the time until activation, the SUEP nonuniversity study sites had the fastest times, with a median time of seven days (Q1: 4, Q3: 33), followed by the HAP study sites with eight days (Q1: 6, Q3: 21.5) and the SUEP university study sites with 14 days (Q1: 6.5, Q3: 27.5). More than half of all study sites (14 SUEP university study sites: 52%; 20 SUEP nonuniversity study sites: 61%; 6 HAP study sites: 55%) were activated within two weeks after approval of their ethics applications.

The HAP study sites had the shortest time from activation until the first patient was recruited with a median time of 29 days (Q1: 21,75, Q3: 43,75), followed by the SUEP university study sites with 38 days (Q1: 13, Q3: 62) and the SUEP nonuniversity study sites with 47 days (Q1: 21,75, Q3: 83,75). Sixty-four percent of the HAP (*n* = 7), 41% of the SUEP university (*n* = 11) and 24% of the SUEP nonuniversity study sites (*n* = 8) started recruiting one month after activation. Altogether, the HAP started recruiting the fastest: two months after approval of their ethics applications, 82% of HAP sites (*n* = 9) recruited patients, followed by 56% of SUEP university sites (*n* = 15) and 27% of SUEP nonuniversity sites (*n* = 9).

### Recruitment performance

During the period of our evaluation, 2,179 patients were recruited by the SUEP and HAP. Of the 75 activated study sites, only 59 recruited patients by the end of 2021, resulting in an average recruitment performance of 37 patients per study site (Table 3). Reasons for nonrecruiting sites were study site dropouts and late activation (nine study sites joined in November and December 2021). When comparing recruitment performance, nonuniversity study sites included fewer patients than university study sites (mean: 11 vs. 53). In the nonuniversity subgroup, outpatient practices recruited fewer patients than nonuniversity hospitals (mean: 7 vs. 16). SUEP university study sites recruited more patients than HAP sites (mean: 53 vs. 43). Furthermore, in the SUEP, the high-performing study sites (*n* = 13) recruited more than half of all SUEP patients. This trend was even more pronounced in the HAP: over two-thirds of patients were recruited by the high-performing study sites (*n* = 3). The low-performing HAP study sites (*n* = 3) recruited only 3% of all HAP patients.

**Table 3:** Recruitment performance of study sites of the Cross-Sectoral Platform (SUEP) and High-Resolution Platform (HAP). The recruited patients and number of study sites are given in absolute numbers. The average recruitment performance per study site is reflected by the ratio of the patient’s recruitment and the number of study sites of the respective platform and health sectors. The number of patients recruited by high-performing (HP) and low-performing (LP) study sites is given in absolute numbers and as a proportion within their group

Platform/Sector	Patients recruited n (%)	No. of sites n (%)	Average recruitment performance	Patients recruited by LP sites n (%)	Patients recruited by HP sites n (%)
**SUEP**	1,707 (78)	48 (81)	36	39 (2)	979 (57)
University sites	1,491 (68)	28 (47)	53	138 (9)	604 (41)
Nonuniversity sites	216 (10)	20 (34)	11	8 (4)	136 (63)
Local hospitals	131 (6)	8 (14)	16	5 (4)	81 (62)
Outpatient practices	85 (4)	12 (20)	7	2 (2)	48 (56)
**HAP**	472 (22)	11 (19)	43	14 (3)	329 (70)
**Total**	2,179 (100)	59 (100)	37	50 (2)	1,269 (58)

**Fig. 7:**
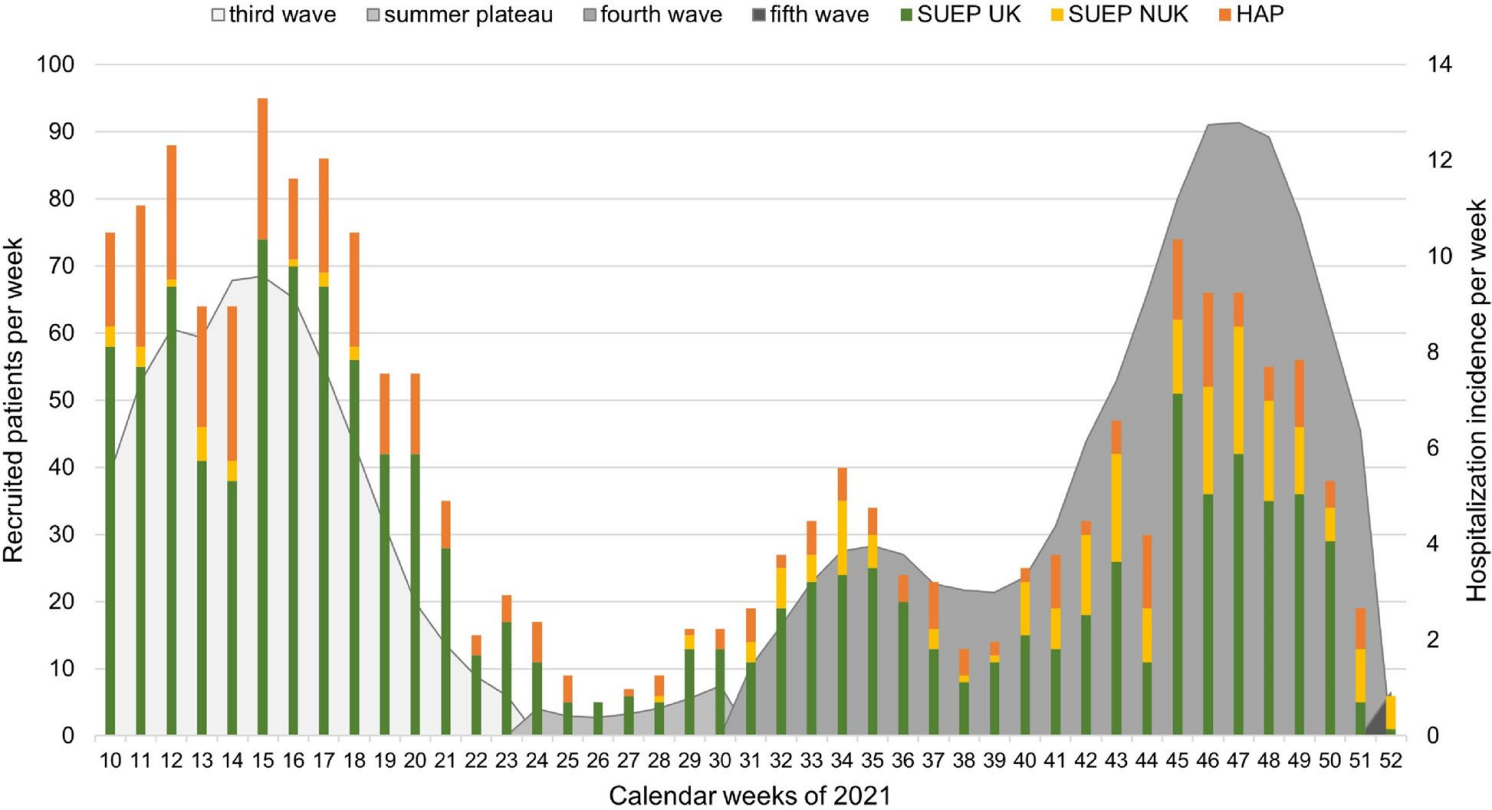
Correlation of hospitalization incidence in Germany and patient recruitment in the Cross-Sectoral Platform (SUEP, university (UK) and nonuniversity (NUK)) and High-Resolution Platform (HAP) study sites. Recruited patients per week are shown as stacked columns. The observation period is reported in calendar weeks. COVID-19 hospitalization incidence represents the mean number of hospitalizations due to COVID-19 infections per 100,000 inhabitants per calendar week in Germany. Waves are classified according to the specifications of the Robert Koch Institute [27] and visualized in different shades

We further wanted to investigate whether the dynamics of the pandemic influenced the patient recruitment rates in NAPKON. During the second wave, the COVID-19 incidence was negatively correlated with the recruitment rate in NAPKON, and only a few study sites were activated. Starting with the third COVID-19 wave in Germany, we observed a highly positive correlation between the number of patients recruited per week and the prevailing incidence as well as the hospitalization incidence (Fig. 7, Fig. S1, Tables S2 and S3). With the spread of the Delta variant in Germany — initiating the start of the fourth wave — incidence and recruitment showed a similar course, but patient recruitment never reached the same peak as before, while COVID-19 incidence far exceeded its earlier maximum (Fig. S1). In contrast, the correlation between hospitalization incidence and patient recruitment remained similar during the third and fourth waves (Fig. 7).

### Willingness to consent

As an indicator of general willingness to consent in the study population, we finally examined the decisions of patients to provide specific consent in the SUEP and the HAP (Table 4). We considered the decisions of 1,683 patients out of 1,707 in the SUEP, since 24 patients (1.4%) had withdrawn their overall consent in the interim and therefore no detailed data were available. Of the 472 HAP patients, the additional consent choices of 450 patients were taken into consideration because 22 patients (4.6%) withdrew their overall consent or were provisionally excluded from the study. The highest refusal rates were for consent to biosample collection and data transfer to non-EU regulation-conforming countries (related to options for the entire study population). The highest approval rates were found for permission to be recontacted for additional requests and data collection from pretreating physicians.

**Table 4:** Specific informed consent options for the Cross-Sectoral Platform (SUEP) and High-Resolution Platform (HAP). The number of patients who agreed or refused to give specific consent was extracted from the trusted third party and reported in absolute numbers and as a proportion of the respondent population. For the SUEP, all options were available to all patients; for the HAP, some options were only available at certain study sites or at certain times, as indicated in the “total” column

	**Specific consent**	**Yes**	**No**	**Total**
**SUEP**	Biosample collection	1,373 (82%)	310 (18%)	1683
	Datatransfer to non-EU countries	1,459 (87%)	224 (13%)	1683
	Recontact for additional requests	1,602 (95%)	81 (5%)	1683
	Recontact for additional findings	1,632 (97%)	51 (3%)	1683
	Data collection from pretreating physicians	1,634 (97%)	49 (3%)	1683
**HAP**	Datatransfer to non-EU countries	388 (86%)	62 (14%)	450
	Recontact for additional findings	422 (94%)	28 (6%)	450
	Recontact for additional requests	439 (98%)	11 (2%)	450
	Genetic testing	431 (96%)	19 (4%)	450
	Additional blood samples for substudy	78 (74%)	28 (26%)	106
	Additional computed tomography	128 (78%)	37 (22%)	165
	Cooperation with industry partners	220 (89%)	27 (11%)	247

## Discussion

Our analysis highlighted some of the major challenges in setting up a multicenter national cohort study during a pandemic in Germany. Of note, in the specific setting of NAPKON as a study with very high resources, considerable public attention, and in a field of great urgency, the median time to a positive ethics vote was less than two weeks, and 30 study sites (65%) were able to join NAPKON within less than three weeks each. In that respect, other than expected, the primary source of recruitment delays was neither the need for ethics consultations nor study activation but on-site organization and resulting lag time between completed study activation and actual inclusion of patients. However, we also documented the high level of bureaucracy and lack of standardization for submission of documents to ethics committees and considerable heterogeneity in votes and annotations, resulting in delays of multiple months for some centers.

The difficulties in clinical trial ethics submission and other clinical study initiation processes have been previously described as challenging. A survey of European hospitals participating in a prospective observational study on chronic postsurgical pain was used to analyze the ethics processes of 24 hospitals in 11 European countries [28]. The approval processes took two weeks to two months due to considerable variation in the approval procedures by European ethics committees. Duley et al. (2008) pointed out challenges in setting up randomized trials [29]. Study initiation processes took approximately one year because study sites had to have their study protocol approved by their own ethics committee even though many other ethics committees had already approved the documents. In comparison to these studies, the application process for ethical approval of the multicenter prospective cohort study NAPKON was fast for most centers. Many ethics committees voted within a few days, giving priority to COVID-19 research projects [30, 31]. Processes were accelerated, for example, by electronic submissions and the possibility of joining the primary vote [32, 33]. However, extreme outliers posed a risk to individual study sites and to the achievement of the overall study objectives. Delayed votes resulted from both waiting for a response from the responsible ethics committee and the need for performing revisions and harmonizing new document versions. Some centers did not meet their site recruitment goal due to their individual late start and consequently had to return grant funds. For affected centers, it was unclear when center initiation could begin. The most extreme delay from ethics approval to first patient recruitment was 170 days. However, some study sites started recruiting the day after ethics approval, as they underwent study activation and concluded all necessary preparations during the ethics application process. In high-urgency scenarios, using the ethics consultation time for study site preparation seems to be a reasonable approach to save time, although retraining may become necessary in case of unexpected delays during the approval process.

Focusing on the votes themselves, we found that the presence of annotations from ethics committees resulted in significantly longer approval processes. In some cases, adjustments were necessary to the master study documents, whereas in others, annotations were resolved by local versions of individual study documents. The heterogeneity in review time and the number of annotations resulting from the first review between ethics committees was striking. Such low interrater reliability may be indicative of either different assessment standards, i.e., which aspects of a submission are taken into consideration and what the threshold is for annotations, or different assessment quality, i.e., how robust the respective assessment standard is implemented. While our study was not suited to differentiate between these both causes, this poses a multifaceted risk for study investigators. On the one hand, they face a considerable chance that a study that has already been accepted and activated at many other German sites may still run into major objections, comprehensive annotations and long delays when initiating the next site. On the other hand, considering that some annotations are deemed sufficiently relevant to change the master study protocol, processes that are too fast and permeable may not offer sufficient protection to patients and investigators.

The annotations were mostly related to “patient information and consent” and “biosample collection.” Harmonization of these documents with cross-site acceptance for both NAPKON and all future studies would be desirable. The Association of Medical Ethics Committees (AKEK) has already developed an electronic tool that assists in the creation of correct patient informed consent documents [34]. In addition, it would be helpful if ethics committees already listed their requirements publicly on the website — or even on the central website of the AKEK — so that they can be met prior to submission to further hasten the processes. The biosample collection requirements are in general transferable to other studies and should therefore be clearly and publicly available on the ethics committees’ websites. The AKEK has already prepared generally applicable recommendations and templates for biosample collection in clinical trials and other studies [35]. These should be adapted to the concerns of all ethics committees. At the European level, the Task Force Research Ethics Committees of the European research infrastructure BBMRI-ERIC was established with the aim of identifying the requirements of international ethics committees with regard to biosample collections, thus facilitating the establishment of multicenter studies [36].

More than a third of received annotations were formal requests, e.g. to print a certain paragraph in bold letters or to frame it. While highlighting defined sections may be important in pointing out key issues to the reader, conflicting format requests eventually amounted to 19 localized versions of patient information and consent documents. In the context of multiple different versions of the master patient information documents and informed consent forms for different settings, situations, and languages, this caused exponential growth in document variants. We were not able to identify meaningful research on the best strategy to visually emphasize important text passages and strongly suggest that the presence of a specific local preference should not be a precondition for ethical clearance.

NAPKON aims to establish a collaborative infrastructure for rapid national performance of key clinical trials, with a special focus on achieving preparedness for future acute public health hazards. Our analyses indicated that recruitment numbers did not depend exclusively on the number of study sites but demonstrated large variance in recruitment performance between the NAPKON sites. While recruitment numbers were correlated with hospitalization incidence, study sites reported that the workload in patient care was at the expense of the study activity. Declining recruitment during the fourth wave of COVID-19 might also have been caused by the diminishing overall attention to the pandemic, resumption of other trial activities related to other diseases, and a shift in the hospitalized COVID-19 population toward vaccination opponents with a lower overall trust level in government-funded research [37, 38]. According to a survey among 6217 health workers by the NUM [39] and a study with 420 participants in Munich [40], one factor that led to a reduced willingness to vaccinate compared to the general population was a migration background. An increased share of patients with language barriers was also an obstacle to successful recruitment, according to informal reports from study centers. This problem occurred even though the patient information documents for the SUEP were translated into eight languages.

In addition to consenting to general participation in the NAPKON study, patients could also agree to additional modules. We found that acceptance of all additional options was generally high, with the highest rejection rates unsurprisingly concerning voluntary invasive procedures. Not only did the European Court of Justice declare the EU-US Privacy Shield invalid on July 16, 2020 [41], but specific consent for data transfer to non-EU countries has also become a requirement for the meaningful exchange of data across borders. Our results, with more than 85% of patients agreeing to share their data with scientists living in countries with less comprehensive data protection laws, demonstrate that most patients are willing to waive enforceable control over their health data in favor of international scientific collaboration.

A systematic literature review including 48 studies about attitudes toward biobanking, broad consent, and data sharing in the US found that patients were more reluctant to share their data if commercial companies were involved [42]. Furthermore, Richter et al. reported on 1,006 participants in a population-based survey from 2019 who agreed to the concept of an anonymous and free of charge “data donation” to third parties for medical research in 78.8% and to universities and public institutions in 96.7%. In contrast, only 16.6% of the participants consented to share their data with industry and private companies as well [43]. Interestingly, 90% of the HAP patients agreed to possible cooperation with industry partners. A possible reason could be the urgency of new research findings and media coverage of vaccine development during the COVID-19 pandemic which may have created greater awareness of the role of industry and private companies in medical research.

Limitations regarding the generalizability of our analysis are that (i) the pandemic affected all processes of study setup and study execution, which may limit comparability with study roll-out in a nonpandemic situation; (ii) willingness to participate and patient consent behavior may have been different than usual for pandemic-related research; (iii) we had no means to ascertain that study centers and coordinating sites actually provided us with all communication among ethics committees, study sites and NAPKON team members; it must be assumed that, e.g., not all telephone calls and emails were recorded and disclosed, and therefore, the overall processing time may have been longer than measured; and (iv) some study sites joined when amendments were already rolled out and the ethics application documents were assessed several times, which may have led to shorter application times and/or fewer annotations.

## Conclusion

With NAPKON, — for the first time in Germany — all university hospitals, as well as many local hospitals and practices, were connected by the common idea of contributing to national and international pandemic responses. Our analysis showed that a rapid build-up is possible if sufficient resources are allocated to the project, especially during a pandemic. Nevertheless, weaknesses of the federated system in Germany were uncovered, which now need to be addressed. Ethics processes need to be further harmonized to avoid hundreds of redundant communications across the many different committees and consolidate assessment standards and review quality. Ethics committees should be encouraged to abstain from enforcing specific local standards regarding formalities (e.g., formatting or exact formulation of specific text passages), which cause extensive work and time delays and have considerable downstream effects, e.g., the need to maintain many different versions of the same documents. In addition, efforts should be taken toward early preparation and robust management of study sites to avoid delays in starting patient enrollment after receiving ethical approval and study site activation.

## Supplementary Information

**Additional file 1:** **Tab. S1.** General overview of documents submitted to the responsible ethics committee for an ethics application for study sites of the Cross-Sectoral Platform (SUEP) and the High-Resolution Platform (HAP). SOP = standard operating procedure; DZHK = German Center for Cardiovascular Research; NUM = Network University Medicine. *submission only at selected ethics committees. **Tab. S2.** Correlation of hospitalization incidence in Germany and patient recruitment in NAPKON Cross-Sectoral Platform and High-Resolution Platform according to the COVID-19 waves defined by the Robert Koch Institute [27]. Wave consideration does not begin until calendar week (KW) 10/2021 because hospitalization incidences were not available until that week. **Tab. S3.** Correlation of hospitalization incidence in Germany and patient recruitment in NAPKON Cross-Sectoral Platform and High-Resolution Platform according to the COVID-19 waves defined by the Robert Koch Institute [27]. Wave consideration begins at calendar week (KW) 45/2020, describing the first week in which NAPKON patients were recruited. **Fig. S1.** Correlation of COVID-19 incidence in Germany and patient recruitment in the Cross-Sectoral Platform (SUEP, university (UK) and nonuniversity (NUK) study sites) and High-Resolution Platform (HAP) study sites. Recruited patients per week are shown as stacked columns. The observation period is reported in calendar weeks, starting with the NAPKON recruitment launch in November 2020 and ending in the last calendar week of 2021. COVID-19 incidence represents the mean of infections per 100,000 inhabitants per calendar week in Germany [26]. Waves are classified according to the specifications of the Robert Koch Institute [27] and visualized in different shades.

## Data Availability

The dataset supporting the conclusions of this article is included within the article and its additional files.

## Abbreviations

AKEK: Arbeitskreis Medizinischer Ethik-Kommissionen / Working Group of Medical Ethics Committees
BBMRI-ERIC: Biobanking and BioMolecular resources Research Infrastructure European Research Infrastructure Consortium
BMBF: Bundesministerium für Bildung und Forschung / Federal Ministry of Education and Research
CANCOV: Canadian COVID-19 Prospective Cohort Study
COVID-19: Coronavirus disease 2019
DZKH: Deutsches Zentrum für Herz-Kreislauf-Forschung e.V. / German Center for Cardiovascular Diseases
FrenchCOVID: French COVID Cohort
HAP: Hochauflösende Plattform / High-Resolution Platform
ISARIC: International Severe Acute Respiratory and emerging Infection Consortium
NAPKON: Nationales Pandemie Kohorten Netz / National Pandemic Cohort Network
NUM: Netzwerk Universitätsmedizin / Network University Medicine
NUK: Nicht-universitäre Klinik / nonuniversity hospital
POP: Populationsbasierte Plattform / Population-Based Platform
RKI: Robert-Koch-Institut / Robert Koch Institute
SARS-Brazil: Brazilian Registry for Clinical Presentation of Individuals With COVID-19
SARS-CoV-2: Severe acute respiratory syndrome coronavirus 2
SOP: Standard operating procedure
SUEP: Sektorenübergreifende Plattform / Cross-Sectoral Platform
UK: Universitätsklinik / university hospital

## References

[1] Isaric clinical characterisation group. Global outbreak research: harmony not hegemony. Lancet Infect Dis. 2020;20(7):770–2. 32502433 10.1016/S1473-3099(20)30440-0 PMC7266570

[2] ClinicalTrials.gov. 2022 [Available from: https://clinicaltrials.gov/.

[3] Cheng ZJ, Shan J. 2019 Novel coronavirus: where we are and what we know. Infection. 2020;48(2):155–63. 32072569 10.1007/s15010-020-01401-y PMC7095345

[4] Wu Z, McGoogan JM. Characteristics of and important lessons from the Coronavirus disease 2019 (COVID-19) outbreak in China: summary of a report of 72314 cases from the Chinese center for disease control and prevention. JAMA. 2020;323(13):1239–42. 32091533 10.1001/jama.2020.2648

[5] Mishra SK, Tripathi T. One year update on the COVID-19 pandemic: where are we now? Acta Trop. 2021;214: 105778. 33253656 10.1016/j.actatropica.2020.105778 PMC7695590

[6] Myoung J. Two years of COVID-19 pandemic: where are we now? J Microbiol. 2022;60(3):235–7. 35235176 10.1007/s12275-022-1679-x PMC8890008

[7] Cancov-Study-Group. CANCOV – The Canadian COVID-19 Prospective Cohort Study 2022 [Available from: https://cancov.net/.

[8] ClinicalTrials.gov. The Canadian COVID-19 Prospective Cohort Study (CANCOV) NCT05125510 202q [Available from: https://clinicaltrials.gov/ct2/show/NCT05125510.

[9] ClinicalTrials.gov. Brazilian Registry for Clinical Presentation of Individuals With COVID-19 (SARS-Brazil) (SARS-Brazil) NCT04479488 2020 [Available from: https://clinicaltrials.gov/ct2/show/NCT04479488.

[10] ClinicalTrials.gov. French COVID Cohort (FrenchCOVID) NCT04262921 2020 [Available from: https://clinicaltrials.gov/ct2/show/NCT04262921.

[11] ISARIC. International Severe Acute Respiratory and emerging Infection Consortium 2022 [Available from: https://isaric.org/research/covid-19-clinical-research-resources/.

[12] Schons M, Pilgram L, Reese J-P, Stecher M, Anton G, Appel KS, et al. The German National Pandemic Cohort Network (NAPKON): rationale, study design and baseline characteristics. Eur J Epidemiol. 2022;37:849. 35904671 10.1007/s10654-022-00896-z PMC9336157

[13] Reinhart K, Welte T. Klinische Studien: Abgehängtes Deutschland. Dtsch Arztebl International. 2022;119(16):A706–7.

[14] Beck C. Zur Weiterentwicklung der deutschen Forschungslandschaft 2016 [Available from: https://www.mpg.de/10357894/zur-weiterentwicklung-der-deutschen-forschungslandschaft.

[15] Pilgram L, Schons M, Jakob CEM, Classen AY, Franke B, Tscharntke L, et al. The COVID-19 pandemic as an opportunity and challenge for registries in health services research: lessons learned from the Lean European Open Survey On SARS-CoV-2 infected patients (LEOSS). Gesundheitswesen. 2021;83(1):45–53. 10.1055/a-1655-8705 34731893

[16] Michalik C, Dress J, Ngouongo S, Staubert S, Weber U, Brockmeyer N, et al. Requirements and tasks of cohorts and registers, the German KoRegIT project. Stud Health Technol Inform. 2014;205:1085–9. 25160356

[17] Schmidt CO, Krabbe CEM, Schossow J, Berger K, Enzenbach C, Kamtsiuris P, et al. Quality standards for epidemiologic cohort studies : An evaluated catalogue of requirements for the conduct and preparation of cohort studies. Bundesgesundheitsblatt Gesundheitsforschung Gesundheitsschutz. 2018;61(1):65–77. 29147857 10.1007/s00103-017-2658-y

[18] Patuleia SIS, Hagenaars SC, Moelans CB, Ausems M, van Gils CH, Tollenaar R, et al. Lessons learned from setting up a prospective, longitudinal, multicenter study with women at high risk for breast cancer. Cancer Epidemiol Biomarkers Prev. 2021;30(3):441–9. 33082203 10.1158/1055-9965.EPI-20-0770

[19] Kates SL, Hurni S, Chen MS. Development and challenges in setting up an international bone infection registry. Arch Orthop Trauma Surg. 2020;140(6):741–9. 31701213 10.1007/s00402-019-03303-7 PMC7202964

[20] Verband der Universitätsklinika Deutschlands. Übersicht der Universitätsklinika in Deutschland 2023 [Available from: https://www.uniklinika.de/die-deutschenuniversitaetsklinika/uebersicht-der-universitaetsklinika/.

[21] Kurth F, Roennefarth M, Thibeault C, Corman VM, Muller-Redetzky H, Mittermaier M, et al. Studying the pathophysiology of coronavirus disease 2019: a protocol for the Berlin prospective COVID-19 patient cohort (Pa-COVID-19). Infection. 2020;48(4):619–26. 32535877 10.1007/s15010-020-01464-x PMC7293426

[22] Bundesärztekammer. (Muster-)Berufsordnung für die in Deutschland tätigen Ärztinnen und Ärzte – MBO-Ä 1997 – in der Fassung des Beschlusses des 124. Deutschen Ärztetages vom 5. Mai 2021 in Berlin. Deutsches Ärzteblatt. 2021;118(23).

[23] Schmidt PDmG. Empfehlung für den Umgang mit multizentrischen Studien außerhalb von AMG oder MPG durch Ethik-Kommissionen, Arbeitskreis Medizinischer Ethik-Kommissionen in der Bundesrepublik Deutschland e.V. 2019 [Available from: https://www.akek.de/wp-content/uploads/Studienprotokolle_Vorlagen_StandJuni2019.docx.

[24] Bayerische Landesärztekammer. Berufsordnung für die Ärzte Bayerns. Bayerisches Ärzteblatt. 2016 [Available from: https://www.bayerisches-aerzteblatt.de/fileadmin/aerzteblatt/spezial/2016/01/komplettpdf/Berufsordnung_5_2016_.pdf.

[25] Smart-Q. ethikPool [Available from: https://www.smart-q.de/ed-portfolio/ethikpool/.

[26] RKI. Robert Koch Institute - Täglicher Lagebericht des RKI zur Coronavirus-Krankheit-2019 (COVID-19) 2022 [2022 Feb 28]. Available from: https://www.rki.de/DE/Content/InfAZ/N/Neuartiges_Coronavirus/Situationsberichte/Gesamt.html.

[27] RKI. Robert Koch Institute - Epidemiologisches Bulletin 10/2022 2022 [Available from: https://www.rki.de/DE/Content/Infekt/EpidBull/Archiv/2022/Ausgaben/10_22.pdf?__blob=publicationFile.

[28] Stamer UM, Naef N, Porz R, Stuber F, Leva B, Meissner W, et al. Ethical procedures and patient consent differ in Europe. Eur J Anaesthesiol. 2015;32(2):126–31. 25503525 10.1097/EJA.0000000000000206

[29] Duley L, Antman K, Arena J, Avezum A, Blumenthal M, Bosch J, et al. Specific barriers to the conduct of randomized trials. Clin Trials. 2008;5(1):40–8. 18283079 10.1177/1740774507087704

[30] Ethikkommission der Medizinischen Fakultät der Universität zu Köln. Hinweis zur Bearbeitungszeit "sonstige Forschung" 2022 [Available from: https://medfak.uni-koeln.de/forschung/forschungsfoerderung/klinische-forschung/ethikkommission/aktuelles.

[31] Ethikkommission bei der Sächsischen Landesärztekammer. Aktuelle Hinweise zu Covid-19 und ihre Auswirkungen 2022 [Available from: https://www.slaek.de/de/01/ethikkommission.php.

[32] Ethik-Kommission der Ärztekammer Westfalen-Lippe und der Westfälischen Wilhelms-Universität. Internetseiten der Ethik-Kommission der Ärztekammer Westfalen-Lippe und der Westfälischen Wilhelms-Universität 2022 [Available from: https://www.aekwl.de/fuer-aerzte/ethik-kommission/.

[33] Ethik-Kommission der Ärztekammer Nordrhein. Aktuelle Informationen für Antragsteller / Sponsoren aufgrund der Covid-19-Pandemie 2022 [Available from: https://www.aekno.de/aerztekammer/ethik-kommission/aktuelles-der-ethik-kommission.

[34] Association of Medical Ethics Committees (AKEK). eTIC – electronic Tool for Informed Consent documents [Available from: https://www.akek.de/en/etic-2/.

[35] Association of Medical Ethics Committees (AKEK). Biobanks [Available from: https://www.akek.de/en/biobanken/.

[36] BBMRI-ERIC. Task Force Research Ethics Committees [Available from: https://www.bbmri-eric.eu/elsi/task-force-research-committee-ethics/.

[37] Polack FP, Thomas SJ, Kitchin N, Absalon J, Gurtman A, Lockhart S, et al. Safety and efficacy of the BNT162b2 mRNA Covid-19 vaccine. N Engl J Med. 2020;383(27):2603–15. 33301246 10.1056/NEJMoa2034577 PMC7745181

[38] Troiano G, Nardi A. Vaccine hesitancy in the era of COVID-19. Public Health. 2021;194:245–51. 33965796 10.1016/j.puhe.2021.02.025 PMC7931735

[39] Schug C, Erim Y, Geiser F, Hiebel N, Beschoner P, Jerg-Bretzke L, et al. Vaccination willingness against COVID-19 among healthcare workers in Germany : results from a university medicine network survey between November 2020 and January 2021. Bundesgesundheitsblatt Gesundheitsforschung Gesundheitsschutz. 2022;65(1):74–85. 34554277 10.1007/s00103-021-03418-6 PMC8458789

[40] Akturk Z, Linde K, Hapfelmeier A, Kunisch R, Schneider A. COVID-19 vaccine hesitancy in people with migratory backgrounds: a cross-sectional study among Turkish- and German-speaking citizens in Munich. BMC Infect Dis. 2021;21(1):1214. 34872525 10.1186/s12879-021-06940-9 PMC8647512

[41] BBC. EU-US Privacy Shield for data struck down by court 2020 [Available from: https://www.bbc.com/news/technology-53418898.

[42] Garrison NA, et al. A systematic literature review of individual’s perspectives on broad consent and data sharing in the United States. Genet Med. 2016;18(7):663–71. 26583683 10.1038/gim.2015.138 PMC4873460

[43] Richter G, Borzikowsky C, Lesch W, Semler SC, Bunnik EM, Buyx A, et al. Secondary research use of personal medical data: attitudes from patient and population surveys in The Netherlands and Germany. Eur J Hum Genet. 2021;29(3):495–502. 33005018 10.1038/s41431-020-00735-3 PMC7940390

