# Supplementary material for: Ethical and coordinative challenges in setting up a national cohort study during the COVID-19 pandemic in Germany
Source: BMC Med Ethics. 2023 Oct 17;24:84. doi: 10.1186/s12910-023-00959-0 (PMC10583323; doi:10.1186/s12910-023-00959-0)
Supplement: Supplementary file 1 — Additional file 1: Tab. S1. General overview of documents submitted to the responsible ethics committee for an ethics application for study sites of the Cross-Sectoral Platform (SUEP) and the High-Resolution Platform (HAP). SOP = standard operating procedure; DZHK = German Center for Cardiovascular Research; NUM = Network University Medicine. *submission only at selected ethics committees. Tab. S2. Correlation of hospitalization incidence in Germany and patient recruitment in NAPKON Cross-Sectoral Platform and High-Resolution Platform according to the COVID-19 waves defined by the Robert Koch Institute [27]. Wave consideration does not begin until calendar week (KW) 10/2021 because hospitalization incidences were not available until that week. Tab. S3. Correlation of hospitalization incidence in Germany and patient recruitment in NAPKON Cross-Sectoral Platform and High-Resolution Platform according to the COVID-19 waves defined by the Robert Koch Institute [27]. Wave consideration begins at calendar week (KW) 45/2020, describing the first week in which NAPKON patients were recruited. Fig. S1. Correlation of COVID-19 incidence in Germany and patient recruitment in the Cross-Sectoral Platform (SUEP, university (UK) and nonuniversity (NUK) study sites) and High-Resolution Platform (HAP) study sites. Recruited patients per week are shown as stacked columns. The observation period is reported in calendar weeks, starting with the NAPKON recruitment launch in November 2020 and ending in the last calendar week of 2021. COVID-19 incidence represents the mean of infections per 100,000 inhabitants per calendar week in Germany [26]. Waves are classified according to the specifications of the Robert Koch Institute [27] and visualized in different shades. [file 12910_2023_959_MOESM1_ESM.pdf]

Ethical and coordinative challenges in setting up a national cohort study during the COVID-19 pandemic in Germany

BMC Medical Ethics

Authors

Katharina Tilch, Sina M. Hopff, Katharina Appel, Monika Kraus, Bettina Lorenz-Depiereux, Lisa Pilgram, Gabi Anton, Sarah Berger, Ramsia Geisler, Kirsten Haas, Thomas Illig, Dagmar Krefting, Roberto Lorbeer, Lazar Mitrov, Maximilian Muenchhoff, Matthias Nauck, Christina Pley, Jens-Peter Reese, Siegbert Rieg, Margarete Scherer, Melanie Stecher, Christoph Stellbrink, Heike Valentin, Christof Winter, Martin Witzenrath and J. Janne Vehreschild

Corresponding author: Katharina Tilch, University of Cologne, Faculty of Medicine and University Hospital Cologne, Department I of Internal Medicine, Center for Integrated Oncology Aachen Bonn Cologne Duesseldorf, tilch.katharina@gmail.com, 0009-0007-0870-0350

Supplementary Information

Tab. S1 General overview of documents submitted to the responsible ethics committee for an ethics application for study sites of the Cross-Sectoral Platform (SUEP) and the High-Resolution Platform (HAP). SOP = standard operating procedure; DZHK = German Center for Cardiovascular Research; NUM = Network University Medicine. *submission only at selected ethics committees

Documents to be submitted SUEP	Documents to be submitted HAP
• Confirmation of insurance<br>• Cover letter ethics application<br>• CV of applicant physician<br>• Data set of the SUEP<br>• Information on electronic recording of electronic consent<br>• Informed consent forms<br>• Insurance policy<br>• Note to file: presumed will<br>• Patient information<br>• Primary ethics vote<br>• Revocation form<br>• Study protocol<br>• SOP Data Transmission<br>• SOP Manual<br>• Table of submitted documents and significant changes<br>• Terms of use of the NUM	• Concept paper of the NUM<br>• Confirmation of insurance*<br>• Cover letter ethics application<br>• CV of applicant physician<br>• Data flow diagram<br>• Data protection concept of the DZHK<br>• Ethics application<br>• Informed consent forms<br>• Insurance policy*<br>• Network diagram of the NUM<br>• Patient information<br>• Primary ethics vote<br>• Study protocol<br>• Table: List of subprojects*<br>• Vote of the State Commissioner for Data Protection and Freedom of Information on the Data Protection Concept of the Trust Agency (Mecklenburg-Western Pomerania)

• Translation certificates<br>• Translations of patient information and consent forms	• Vote of the State Commissioner for Data Protection on the data protection concept of the DZHK (Lower Saxony)

Tab. S2 Correlation of hospitalization incidence in Germany and patient recruitment in NAPKON Cross-Sectoral Platform and High-Resolution Platform according to the COVID-19 waves defined by the Robert Koch Institute (25). Wave consideration does not begin until calendar week (KW) 10/2021 because hospitalization incidences were not available until that week.

	Pearson correlation coefficient	95%-confidence interval	p value
**All waves (KW10/21-52/21)**	0.77	0.61-0.87	< 0.001
**Third wave (KW10/21-23/21)**	0.88	0.65-0.96	< 0.001
**Summer plateau (KW24/21-30/21)**	0.74	-0.02-0.96	0.055
**Fourth wave (KW31/21-51/21)**	0.86	0.68-0.94	< 0.001

Tab. S3 Correlation of hospitalization incidence in Germany and patient recruitment in NAPKON Cross-Sectoral Platform and High-Resolution Platform according to the COVID-19 waves defined by the Robert Koch Institute (25). Wave consideration begins at calendar week (KW) 45/2020, describing the first week in which NAPKON patients were recruited.

	Pearson correlation coefficient	95%-confidence interval	p value
**All waves (KW45/20-52/21)**	0.31	0.06-0.52	0.017
**Second wave (KW45/20-08/21)**	-0.84	-0.94-0.60	< 0.001
**Third wave (KW09/21-23/21)**	0.88	0.67-0.96	< 0.001
**Summer plateau (KW24/21-30/21)**	0.63	-0.22-0.94	0.126
**Fourth wave (KW31/21-51/21)**	0.83	0.61-0.93	< 0.001

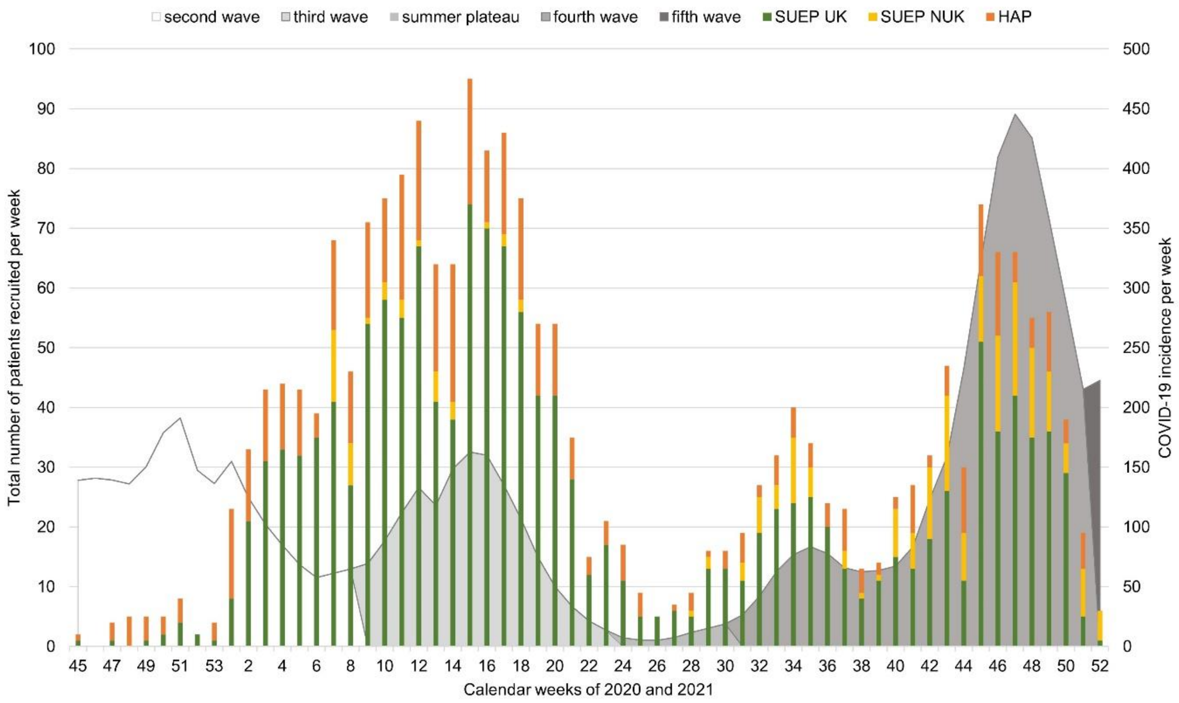

Fig. S1 Correlation of COVID-19 incidence in Germany and patient recruitment in the Cross-Sectoral Platform (SUEP, university (UK) and nonuniversity (NUK) study sites) and High-Resolution Platform (HAP) study sites. Recruited patients per week are shown as stacked columns. The observation period is reported in calendar weeks, starting with the NAPKON recruitment launch in November 2020 and ending in the last calendar week of 2021. COVID-19 incidence represents the mean of infections per 100,000 inhabitants per calendar week in Germany (24). Waves are classified according to the specifications of the Robert Koch Institute (25) and visualized in different shades.
